# Serology, virulence and molecular characteristics of *Vibrio parahaemolyticus* isolated from seafood in Zhejiang province

**DOI:** 10.1371/journal.pone.0204892

**Published:** 2018-10-04

**Authors:** Xiao Chen, Qiaoyun Zhu, Fei Yu, Wen Zhang, Ruonan Wang, Xianfei Ye, Linfeng Jin, Yanchao Liu, Shufei Li, Yu Chen

**Affiliations:** 1 State Key Laboratory for Diagnosis and Treatment of Infectious Diseases, Collaborative Innovation Center for Diagnosis and Treatment of Infectious Diseases, the First Affiliated Hospital, School of Medicine, Zhejiang University, Hangzhou, Zhejiang, China; 2 Key Laboratory of Clinical In Vitro Diagnostic Techniques of Zhejiang Province, Department of Clinical Laboratory, the First Affiliated Hospital, School of Medicine, Zhejiang University, Hangzhou, Zhejiang, China; Universidad Nacional de la Plata, ARGENTINA

## Abstract

*Vibrio parahaemolyticus* is a leading foodborne pathogen in southeastern China. In this study, 105 strains of *V*. *parahaemolyticus* were isolated from fresh seafood in 2013 and 2014. The serotypes, virulence-associated genes and sequence types (STs) of these strains were analyzed. 26 defined serotypes were identified and 69 strains (65.7%) had untypeable O or K antigen. 8 strains (7.6%) had the virulence-associated gene *tdh* and no strain carried the *trh* gene. 45.7% (48/105) of isolates contained all four T3SS1 genes and 50% (4/8) *tdh*^*+*^ *trh*^*-*^ *V*. *parahaemolyticus* isolates lacked at least one of the four tested T3SS2α genes. 105 strains could be categorized into 84 STs and only 3 STs (ST3, ST8, ST675) had appeared in clinical strains. *V*. *parahaemolyticus* strains from seafood have more diverse and untypeable serotypes, less virulence-associated genes and more STs than strains from clinical sources.

## Introduction

*Vibrio parahaemolyticus* is a halophilic bacterium that is naturally present in marine and estuarine environments and has been isolated from the coastal regions of most continents [1–3]. Since it was first identified as a causative agent of gastroenteritis in Japan in 1950, *V*. *parahaemolyticus* had been recognized as a common contaminant of seafood and was responsible for acute diarrheal illness in human [4]. In many Asian countries, such as China, Japan and Taiwan, approximately half of foodborne illnesses are caused by *V*. *parahaemolyticus* [5]. In addition, *V*. *parahaemolyticus* outbreaks are frequently reported in the United States [6–7]. Even in Europe, sporadic outbreaks are consistently reported in coastal countries such as Spain, Italy and Norway [8–9].

The presence of *tdh* and/or *trh* genes is associated with the ability of a strain to cause gastroenteritis. Up to 90% of clinical isolates possess *tdh* and/or *trh* gene. Since 1996, O3:K6 serotype of *V*. *parahaemolyticus* became prevalent in the world, causing pandemics [4, 10]. Group-specific PCR (GS-PCR) method can distinguish the O3:K6 strains isolated before and after 1996 [11]. The pandemic clone had characteristics of *tdh*^*+*^, *trh*^*-*^, *toxRS/new*^*+*^ (a unique *toxRS* sequence detectable by GS-PCR) and *orf8*^+/-^ (the *orf8* sequence of f237 phage) [12]. Recent study showed that the O3:K6 pandemic clone was still predominant among clinical isolates [7, 13–14].

Type III secretion system (T3SS) has also been shown to be associated with the pathogenicity of *V*. *parahaemolyticus*. Previous studies demonstrated that T3SS1 genes were present in all *V*. *parahaemolyticus* strains; T3SS2α genes were present and co-regulate with *tdh* genes, whereas T3SS2β genes were generally only found in *trh* strains on a pathogenicity island (Vp-PaI) [3, 15].

We previously determined the serology, virulence, antimicrobial susceptibility and molecular characteristics of clinical *V*. *parahaemolyticus* strains in southeastern China from 2009–2013 [13]. However, the characteristics of environmental *V*. *parahaemolyticus* are rarely known. In this study, the serotypes, virulence-associated genes and sequence types (STs) of 105 strains of *V*. *parahaemolyticus* isolated from seafood in 2013 and 2014 in Zhejiang province were analyzed.

## Materials and methods

### Sample collection and microbiological analysis

Fresh seafood samples (oysters, clams, shrimps, and others) were collected from 13 markets in different parts of Zhejiang Province from June to October in 2013 and 2014 (S1 Table). The seafood samples were transported to the laboratory on ice in 2 hours and processed immediately upon receipt. The sample preparation and pathogens isolation procedures had been described in previous studies [16]. The seafood was washed with running potable water and opened aseptically. Twenty-five grams of intra valvular liquid and meat were collected in a sterile jar and combined with 225 ml of 3% alkaline peptone water (APW) at a 1:10 dilution, and the mixture was blended for 60s at 8000 rpm in a Stomacher blender. This broth was then incubated at 35°C ± l°C for 8–12 hours. The enriched broth was subcultured onto selective thiosulfate citrate bile sucrose (TCBS) agar, and the medium was incubated for 16-18h at 35°C ± l°C. Then, presumptive strains (green colonies on TCBS medium) were identified at the species level using the VITEK2 Compact automated microbial identification system (BioMerieux, Marcy-l'Étoile, France).

### Serotyping

O and K antigen assessment was performed using an agglutination test with 11 O (lipopolysaccharide) and 65 K (capsule) antisera (Denka Seiken Ltd., Tokyo, Japan) according to the instructions provided with the reagents. One serotype was defined as a unique combination of O and K serogroups.

### Detection of virulence-associated genes

All *V*. *parahaemolyticus* isolates were tested for the presence of a species-specific marker (*tlh*), and the hemolysin genes (*tdh*, *trh*) by real-time multiplex PCR [17]. Real-time PCR was performed in an Applied Biosystems 7500 (Applied Biosystems, CA, USA). The optimal cycling parameters consisted of a 95°C hold for 60s for the initial denaturation and activation of the hot-start *Taq* polymerase, followed by 45 cycles of amplification, with each cycle consisting of denaturation at 95°C for 5s and a combined primer annealing/extension step at 59°C for 45s.

PCR assays to amplify the pandemic markers were performed using specific primers previously reported to detect *toxRS/new* [11] and *orf8* sequence [18]. The PCR cycling parameters for these assays were as follows: for the *toxRS/new* gene, initial denaturation at 94°C for 3 min, followed by 25 cycles of 30s at 94°C, 30s at 45°C, and 1 min at 72°C, with a final extension of 5 min at 72°C, and for the *orf8* gene, denaturation at 96°C for 5 min, followed by 25 cycles of 1 min at 94°C, 1 min at 53°C, and 1 min at 72°C, with a final extension of 7 min at 72°C.

The isolates were also tested for the type III secretion system (T3SS) genes [19]. The presence of T3SS1 genes (VP1670 [*vscP*], VP1686 [*vopS*], VP1689 [*vscK*], and VP1694 [*vscF*]) was tested using a conventional multiplex PCR. The cycling parameters were as follows: denaturation at 94°C for 2 min, followed by 33 cycles of 45s at 94°C, 40s at 60°C, and 45s at 72°C, with a final extension of 7 min at 72°C. The presence of T3SS2α genes (VPA1362 [*vopB2*], VPA1339 [*vscC2*], VPA1335 [*vscS2*], and VPA1327 [*vopT*]) was tested using a conventional multiplex PCR. The cycling conditions were described for the T3SS1 screening. For the detection of T3SS2β genes (*vscC2*, *vopB2*, *vopC*, and *vscS2*), a conventional multiplex PCR was conducted. The cycling conditions were as follows: denaturation at 94°C for 2 min, followed by 30 cycles of 30s at 94°C, 30s at 55°C, and 2 min at 72°C, with a final extension of 7 min at 72°C.

The pathogenic group was defined as *tdh*^+^ and/or *trh*^+^, and all other isolates were assigned to the non-pathogenic group. The isolates in the pandemic group were defined as *tdh*^*+*^, *trh*^*-*^, *toxRS/new*^*+*^ and *orf8*^*+/*-^, and all other isolates were assigned to the non-pandemic group [7].

### Multilocus sequence typing (MLST)

MLST was performed using the Achtman typing scheme (http://pubmlst.org/vparahaemolyticus/) according to the protocols published on the website. Seven house-keeping genes (*dnaE*, *gyrB*, *recA*, *dtdS*, *pntA*, *pyrC* and *tnaA*) were selected for PCR amplification and sequencing. Alleles at the seven loci were used to identify a unique ST. The STs were subdivided into clonal complexs (CCs), groups, and singletons using eBURST v3.0. BioNumerics v.6.01 (http://www.applied-maths.com) was used to generate a minimum spanning tree (MST) based on the non-concatenated sequences of 7 alleles.

## Results

### Serotypes

105 out of 273 (38.5%) seafood samples (oysters, clams, shrimps, and others), collected from 8 seafood retail markets and 5 supermarkets in Zhejiang province from June to October in 2013 and 2014, were positive for *V*. *parahaemolyticus*. 26 defined serotypes were identified and 69 strains (65.7%) had untypeable for O or K antigen. In these 105 strains, OUT:KUT was the most common serotype (16.2%, 17/105), followed by O3:KUT (7.6%, 8/105), O4:KUT (7.6%, 8/105), O5:KUT (7.6%, 8/105), OUT:K28 (7.6%, 8/105) and O3:K6 (3.8%, 4/105) (Table 1). In our previous study [13], 501 clinical strains collected from Zhejiang province in 2009 to 2013 had 21 serotypes, only 4.0% (20/501) were untypeable O or K antigen, and O3:K6 was the most common serotypes (65.1%, 326/501). So, serotypes of *V*. *parahaemolyticus* from seafood were more diverse and untypeable than from diarrhea patients.

**Table 1:** Distribution of virulence-associated genes, serotypes and STs in *V*. *parahaemolyticus* strains isolated from seafood from June to October in 2013 and 2014.

Hemolysin Genes	Pandemic Markers	T3SS1				T3SS2α				Serotypes
*tdh*	GS-PCR	*vscP*	*vopS*	*vscK*	*vscF*	*vopB2*	*vscC2*	*vscS2*	*vopT*	
+	+	+	+	+	+	-	+	+	+	O3:K6, OUT:K24
+	+	+	+	+	-	+	+	+	+	O3:K6
+	-	+	+	+	+	+	+	+	+	O3:K6
+	-	+	+	+	+	-	+	+	-	O4:K29, O8:KUT
+	-	+	-	+	+	+	+	+	+	O3:K6, OUT:K33
-	-	+	+	+	+	-	+	+	+	O1:K33, OUT:K28
-	-	+	+	+	+	-	+	-	+	O4:K34, OUT:K28
-	-	+	+	+	+	-	+	-	-	O4:K13, OUT:KUT, OUT:KUT
-	-	+	+	+	+	-	-	+	-	OUT:KUT
-	-	+	+	+	+	-	-	-	+	O3:KUT
-	-	+	+	+	+	-	-	-	-	O1:K6, O1:K33, O1:K36, O2:KUT, O3:K5, O3:K28, O3:K31, O3:KUT, O3:KUT, O3:KUT, O3:KUT, O4:K34, O4:KUT, O4:KUT, O5:K20, O5:KUT, O5:KUT(n = 2), O6:K41, O11:K3, O11:K28, OUT:K20, OUT:K28, OUT:K28, OUT:K33, OUT:K24, OUT:K28, OUT:KUT, OUT:KUT, OUT:KUT, OUT:KUT, OUT:KUT, OUT:KUT, OUT:KUT
-	-	+	+	+	-	-	-	-	-	O2:K26, O3:KUT, O3:KUT, O4:K3(n = 2), O4:K4, O4:K15, O4:KUT, O4:KUT, O5:KUT, O5:KUT, OUT:K6, O11:KUT, OUT:K6, OUT:K19, OUT:K30, OUT:K68, OUT:KUT, OUT:KUT
-	-	+	+	-	+	-	-	-	+	OUT:K33
-	-	+	+	-	+	-	-	-	-	O1:K5, O1:K6, O5:KUT, O8:K3, OUT:K24, OUT:K28, OUT:KUT, OUT:KUT
-	-	+	+	-	-	-	-	-	-	O4:KUT, O5:KUT, OUT:KUT
-	-	+	-	+	+	-	+	+	+	O3:K56
-	-	+	-	+	+	-	+	+	-	O3:K21
-	-	+	-	+	+	-	+	-	-	O5:K20, OUT:K48
-	-	+	-	+	+	-	-	-	-	O1:K28, O2:KUT, O3:K28, O3:KUT, O4:K9, O4:KUT, O8:KUT, OUT:K28, OUT:K52, OUT:KUT
-	-	+	-	+	-	-	-	-	-	O1:K46, O3:K31, O5:KUT, OUT:K28, OUT:KUT, OUT:KUT
-	-	+	-	-	+	-	-	-	+	O4:KUT
-	-	+	-	-	+	-	-	-	-	O4:KUT
-	-	-	+	+	+	-	-	-	-	O5:K28

### Distribution of virulence-associated genes

The virulence-associated genes of *V*. *parahaemolyticus* are thermostable direct hemolysin (*tdh*) and thermostable direct hemolysin-related hemolysin (*trh*). The thermolabile hemolysin (*tlh*) is considered a signature molecular marker for *V*. *parahaemolyticus*. All strains from seafood had *tlh* gene but none had *trh* gene (Table 1). Only 8 seafood isolates (7.6%) were positive for *tdh*. The ratio was much lower compared to clinical strains (93%, 466/501) [13]. The serotypes of these 8 *tdh*^+^ strains were O3:K6 (n = 4), O8:KUT (n = 1), O4:K29 (n = 1), OUT:K24 (n = 1) and OUT:K33 (n = 1), and the latter three serotypes did not appeared in clinical strains from Zhejiang province. In these 8 *tdh*^+^ strains, 3 strains (O3:K6 n = 2, OUT:K24 n = 1) were pandemic clone as *tdh*^*+*^, *trh*^*-*^ and *toxRS/new*^*+*^. The *tdh*^-^ *trh*^−^strains with serotypes O1:K36 (n = 1), O3:KUT (n = 8), O4:K4 (n = 1), O5:KUT (n = 8) and O11:KUT (n = 1) were also isolated from diarrhea patients in Zhejiang province during the same period, the difference was *tdh*^-^*trh*^−^clinical strains contained all four T3SS2α genes but not all four genes were detected in *tdh*^-^*trh*^−^seafood isolates (Table 1). In addition, only 50% (4/8) *tdh*^*+*^ *trh*^*-*^ isolates from seafood contained all four T3SS2α genes and 45.7% (48/105) seafood isolates contained all four T3SS1 genes, whereas these proportions of clinical isolates were up to 96% [13]. *V*. *parahaemolyticus* in seafood contained fewer virulence-associated genes.

### Multilocus sequence typing (MLST) analysis

**Fig 1:**
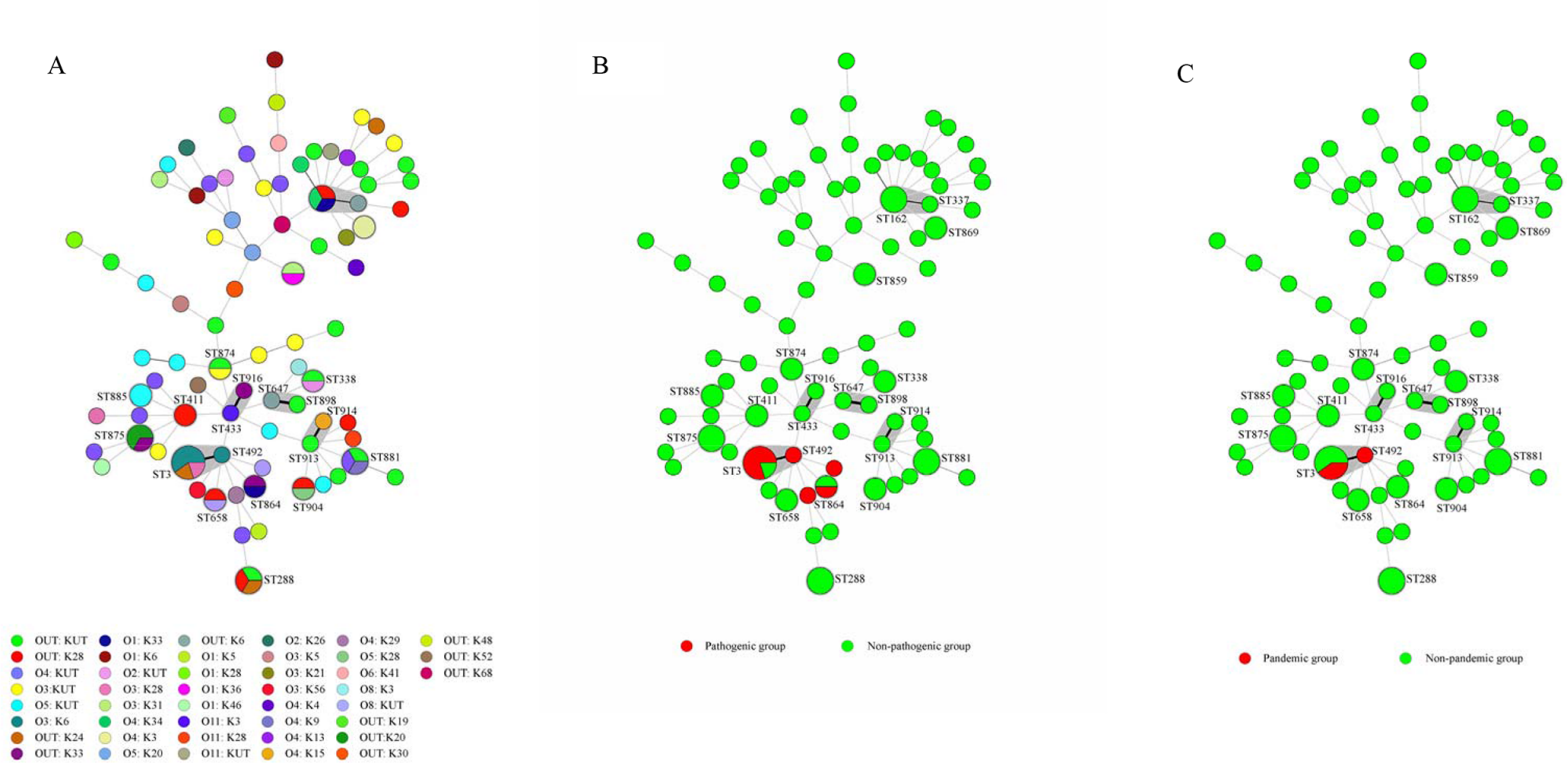
Minimum spanning tree (MST) analysis of 105 seafood *V*. *parahaemolyticus* strains based on MLST data. Each circle corresponds to a ST, and the size of each circle corresponds to the number of isolates. The relationships between strains are indicated by the lengths of the linking branches. Different colors indicate different serotypes and the number of isolates is expressed in brackets. Three MST figures were generated separately based on the available subtyping information. (A) ST *vs* serotypes; (B) ST *vs* pathogenic / non-pathogenic strains; (C) ST *vs* pandemic / non-pandemic strains.

All the 105 strains from seafood were analyzed by multilocus sequence typing (MLST) and categorized into 84 STs (Fig 1). The most frequently observed ST was ST3 (5/105, O3:K6 n = 3, OUT:K24 n = 1, O3:K8 n = 1), being consistent with clinical strains, but only 3 STs (ST3, ST8, ST675) had appeared in clinical strains among 84 STs. The 8 seafood *tdh*^+^ strains (pathogenic group) were categorized into 5 STs (Fig 1B), belonging to ST3 (n = 4), ST8 (n = 1), ST492 (n = 1), ST864 (n = 1) and ST901 (n = 1) respectively. The 3 pandemic strains (*tdh*^*+*^, *trh*^*-*^, *toxRS/new*^*+*^) isolated from seafood belonged to ST3 (n = 2, O3:K6 and OUT:K24) and ST492 (n = 1, O3:K6) (Fig 1C). Only one in 105 strains from seafood had the characteristic of *tdh*^+^ *trh*^−^*toxRS/new*^*+*^ O3:K6 ST3 which was the characteristic of most strains from diarrhea patients.

## Discussion

*V*. *parahaemolyticus* is naturally present in coastal waters. There is an increase in the number of *V*. *parahaemolyticus* infections during the summer and autumn [12–13]. Therefore, we chose the period from June to October to undertake sample collection and analyzed the characteristics (serotypes, virulence-associated genes and STs) of 105 strains from seafood in Zhejiang province.

34.3% of the isolates exhibited defined serotypes, and the agglutination rate was significantly lower than that of the clinical group (93.2%) (χ2 = 213.666, *p*< 0.001). Of the defined serotypes, the isolates from seafood were highly diverse with 26 identified types and no single dominant serotype. 3 strains (O3:K6 n = 2, OUT:K24 n = 1) had pandemic clone characteristics (*tdh*^+^, *trh*^-^, *toxRS/new*^+^) and OUT:K24 serotype was the first reported. Similarly, there have been previous reports that many serotypes strains from environment have the pandemic clone characteristics, such as O3:K6, O1:KUT and O1:K25 in Jiangsu from 2006 to 2014 [18], O3:K6, O3:KUT and O10:KUT in Northwest Mexico between 2004 and 2010 [19], and O2:KUT, O3:KUT in Italy from 2000 to 2010 [9]. Although the incidence of environmental pandemic strains is low, the potential outbreak risk should be closely monitored.

The presence of the *tdh* and/or *trh* gene is associated with the ability of a strain to cause gastroenteritis. In molecular epidemiological studies, up to 90% of clinical isolates possess the *tdh* and/or *trh* gene, whereas their presence in environmental isolates is rare [4, 12–13, 19–21]. However, previously reported environmental isolate data have yielded different results. In a study conducted in Northwest Mexico, pathogenic strains (*tdh*^+^ and/or *trh*^+^) of *V*. *parahaemolyticus* accounted for 62.5% of the environmental isolates [7]; in North America, 79% of isolates from oyster were pathogenic [20]; in Malaysia, 6.5% of isolates from shellfish were pathogenic [2]; in this study, 7.6% of isolates from seafood were pathogenic. The distributions of *tdh* and *trh* vary among geographic regions and time periods. This phenomenon may be related to the marine ecological environment.

The type III secretion system (T3SS) has also been shown to be associated with the pathogenicity of *V*. *parahaemolyticus*. In this study, 54.3% of seafood *V*. *parahaemolyticus* isolates lacked at least one of the four tested T3SS1 genes, and 50% of *tdh*^*+*^ *trh*^*-*^ *V*. *parahaemolyticus* isolates lacked at least one of the four tested T3SS2α genes. The absence of specific T3SS1 and T3SS2α genes has also been observed in *V*. *parahaemolyticus* isolates from oysters [20]. Thus, the horizontal movement of Vp-PaIs between isolates is potentially incomplete. Alternatively, there may be sequence variations in some of these genes that prevented their detection by PCR in this study. Noriea *et al*. suggested that the VPA1362 [*vopB2*] gene of T3SS2α may be a more reliable predictor of virulence than *tdh*, based on *vopB2* found in all *tdh*^*+*^ *trh*^*-*^ clinical strains but not in any of the 130 environmental strains [15]. In our study, the *vopB2* gene was also amplified in all *tdh*^+^ *trh*^-^ clinical isolates, but only 50% (4/8) of *tdh*^+^ *trh*^-^ seafood isolates lacked *vopB2* which was different than Noriea’s study. Surprisingly, several *tdh*^-^ *trh*^-^ isolates contained certain T3SS2 genes but not *vopB2*. These results warrant further exploration. The data related to the distribution of the T3SS genes among environmental strains have not been previously generated in Zhejiang province. This study provides novel information regarding the abundance and characteristics of potential pathogenic *V*. *parahaemolyticus* in this setting.

Previous MLST-based studies have demonstrated that the *V*. *parahaemolyticus* population can be extremely diverse, even within a single geographic locality [13, 22–23]. The eBURST algorithm revealed that the 84 STs in this study belong to 2 clonal complexes, 17 groups and 61 singletons. This discovery implies the general absence of a linkage between STs and supports the hypothesis that these complexes, groups and singletons are genetically exclusive groups. ST3 is largely clinical and representative of pandemic clones in Asia and America [13, 24–25]. In this study, in consistence with previous observations, ST3 was the most common among both clinical (82.6%) and seafood (4.8%) strains and accounted for 90.9% and 66.7% of isolates in the clinical and seafood pandemic groups, respectively. Previously, correlation have been observed between different serogroups and STs, specifically O4 and ST36, O1 and ST417, O6 and ST50, and some STs contained several different serogroups, such as ST43 (O1 and O4), ST3 (O1 and O3) and ST8 (O1 and O4) [13–14]. This phenomenon was observed among the seafood strains in this study, with ST3 being associated with O3:K6, O3:K28 and OUT:K24. Thus, O and K antigen encoding loci appear to be subject to an exceptionally high rate of recombination [15, 26]. This is the first report demonstrating the population structure of seafood *V*. *parahaemolyticus* in Zhejiang province by MLST.

Compared to the clinical isolates in this region [13], the seafood isolates presented more serotypes and a lower proportion of pathogenic and pandemic strains. Certain virulence-associated serotypes and virulence factors were found in both clinical and seafood isolates. The results are in agreement with those of other studies [19–20, 24, 27–28] and imply that there is an association, albeit low, between environmental *V*. *parahaemolyticus* strains and gastroenteritis. Most environmental strains are likely cleared by gastric acid, bile or other host immune defense. However, some serotyes, especially those that harbor virulence factors, can cause human illness. It is also suggested that recombinational exchange has occurred more frequently in the human intestinal tract than in the environment [24]. Carriage of different strains of *V*. *parahaemolyticus* within the human intestinal tract may lead to the emergence of new, potentially pathogenic strains.

In summary, the serotypes and genetic diversity of *V*. *parahaemolyticus* were evaluated, and the pandemic O3:K6 clone was found to be present in the environment, although its incidence was low. The presence and persistence of pathogenic and pandemic environmental *V*. *parahaemolyticus* strains are a matter of concern for public health authorities because the potential for outbreaks along the southeastern China coastline is now well established. Therefore, the information presented in this study can serve as a reference for public health agencies.

## Supporting information

S1 Table The markets name of seafood samples collected. (DOCX)

S2 Table The information of 105 *Vibro parahaemolyticus* strains. (XLSX)
