# Supplementary material for: Serology, virulence and molecular characteristics of Vibrio parahaemolyticus isolated from seafood in Zhejiang province
Source: PLoS One. 2018 Oct 4;13(10):e0204892. doi: 10.1371/journal.pone.0204892 (PMC6171872; doi:10.1371/journal.pone.0204892)
Supplement: S1 Table — (DOCX) [file pone.0204892.s001.docx]

S1 Table. The markets name of seafood samples collected.

| City | Markets name |
| --- | --- |
| Hangzhou | Jinjiang aquatic agricultural products comprehensive market |
|  | Zhongjiang farmer's market |
|  | Hangzhou tide farmer's market |
|  | Xinhua road farmer's market |
|  | Yanjianong farmer's market |
|  | Three fort comprehensive market |
|  | Zhaohui second district farmer's market |
| Ningbo | Ningbo aquatic products trading center |
|  | Jiangdong aquatic products trading center |
|  | Sangjia farmer's market |
|  | Huayan vegetable market |
|  | Haiyi green aquatic products market |
|  | Gangyu aquatic products market |
